# Health Outcomes of U.S. Active Component Service Members Working in High Risk Occupations for Blast Overpressure Exposure: A Retrospective Cohort Study

**Published:** 2026-09-20

**Authors:** Jordan E. Gildehaus, Carlie N. Skellington, Symone M. Baker Miller, Chiping Nieh, Shauna L. Stahlman

**Affiliations:** Department of Preventive Medicine, Madigan Army Medical Center, Joint Base Lewis–McChord, WA: CPT Gildehaus, CPT Skellington; Armed Forces Health Surveillance Division, Public Health Directorate, Defense Health Agency, Silver Spring, MD: Dr. Stahlman, LCDR Baker Miller, Dr. Nieh

## Abstract

Active component military service members are exposed to increased risk of occupational blast overpressure (BOP) exposure during deployment as well as training. This retrospective cohort study examined the incidence of first-time diagnoses of traumatic brain injury (TBI), insomnia, migraine headache, depressive or anxiety disorders, noise-induced hearing injury (NIHI), and essential hypertension among active component service members (ACSMs) in the U.S. Army, Air Force, and Marine Corps. Incidence rates were compared between ACSMs who served in occupations at high risk for BOP exposure (n=831,050) and those who never served in such occupations (n=2,229,594). Overall incidence of TBI was 243.8 per 10,000 person-years in the high-risk occupational cohort and 140.9 per 10,000 person-years in the low-risk occupational cohort. Incidence of NIHI was 342.7 and 218.1 per 10,000 person-years in the high- and low-risk cohorts, respectively. After adjusting for demographic and military characteristics, ACSMs in occupations with high risk of BOP experienced higher incidence rates of TBI (adjusted incidence rate ratio [aIRR] 1.43; 95% CI 1.42, 1.44) and NIHI (aIRR 1.39; 95% CI 1.38, 1.40). Increasing time since entering the cohort was associated with a greater increase in migraine headache incidence among ACSMs in high-risk BOP occupations compared with those in low-risk occupations. Despite these differences, the distribution of disease burden categories from 2024 health care encounters was similar among cohorts. These findings emphasize the importance of mitigating occupational blast hazards and strengthening surveillance efforts to protect the long-term health of ACSMs.

Blast injuries are a significant occupational hazard in the military as well as law enforcement, mining, construction, and chemical manufacturing environments. ^1^ During an explosive event, rapid release of energy generates a primary blast wave that compresses the surrounding air and produces a sudden increase in atmospheric pressure above ambient levels. The positive-pressure phase of the blast wave—termed blast overpressure (BOP)—produces an outward-propagating shock wave, followed by blast wind and a subsequent negative-pressure phase. ^2^

Blast exposure varies by intensity and exposure pattern. Low-level blast exposure is commonly associated with routine weapons training, including firing of heavy-caliber weapons that generate “blast wind” and are often repetitive and cumulative in nature. In contrast, high-level blast exposure is typically associated with acute, high-energy events such as improvised explosive devices, rocket-propelled grenades, and landmines, which generate larger shockwaves and are more often singular or episodic. BOP exposure is therefore characterized not only by intensity (low vs. high) but also by exposure frequency (single vs. repetitive) and operational context (training-related vs. combat-related).

**What are the new findings?**

Adjusted incidence rates of traumatic brain injury (TBI) and noise-induced hearing injury (NIHI) were higher among service members in occupations at high risk for BOP exposure than among those who had never worked in such occupations. Increasing time in the cohort was associated with a greater increase in migraine headache incidence among service members in high-risk BOP occupations than among those in low-risk occupations.

**What is the impact on readiness and force health protection?**

This analysis aimed to characterize potential long-term health consequences associated with serving in military occupations at high risk for BOP exposure and inform force health protection efforts. Enhanced BOP exposure monitoring, occupational hazard mitigation, and targeted health surveillance may help identify and reduce adverse health outcomes among personnel serving in occupations with higher risk.

Active component service members (ACSMs) are at increased risk of occupational BOP exposure during both garrison training and deployment. A retrospective cohort study conducted by the Department of Veterans Affairs found that more than 70% of veterans who served during operations Iraqi Freedom and Enduring Freedom reported histories of blast exposure, most of which involved high-level blast events. ^3 , 4^

Since direct measures of cumulative blast exposure are often unavailable, military occupational specialty serves as a validated proxy, with demonstrated utility in identifying service members with a history of occupational blast exposure. ^5^ Primary military occupational specialties (PMOSs) at greatest risk include armor, artillery, gunnery, combat engineering, explosive ordnance disposal, infantry, medical personnel assigned to expeditionary units, military training instructors, and special operations forces. ^6^

According to the U.S. Department of War Brain Injury Research Coordinating Office, acute BOP exposure exceeding 4 pounds per square inch (psi) may result in adverse health and cognitive effects and has served as the DOW’s working exposure safety threshold since 2022. ^7^ Low- and high-level blast exposures can cause injury through 5 mechanisms of injury: primary blast injury (i.e., overpressure wave), secondary blast injury (i.e., debris), tertiary blast injury (i.e., body displacement), quaternary blast injury (i.e., heat and toxic exposures), and quinary blast injury (i.e., environmental contaminants). ^8^ Clinically, primary blast injury is often associated with symptoms such as poor concentration, dizziness, memory impairment, headache, tremor, noise sensitivity, and tinnitus. ^9 , 10^

Physiologically, the overpressure wave generated during blast events is thought to induce neurovascular inflammation, oxidative stress, vascular injury, and systemic inflammatory responses that may propagate through interconnected biologic pathways. ^11^ Emerging literature further suggests that blast exposure may disrupt blood–brain barrier integrity and contribute to immune and gastrointestinal dysregulation. ^12^ The organ systems most susceptible to primary blast forces are those containing air- or fluid-filled structures, including the central nervous system (e.g., traumatic brain injury [TBI]), ^13 – 16^ auditory system (e.g., tympanic membrane rupture), ^17 , 18^ ocular system (e.g., globe rupture), ^19^ pulmonary system (e.g., “blast lung”), ^20^ and gastrointestinal system (e.g., hemorrhage, perforation). ^21^ Growing evidence indicates additional, less well-characterized health outcomes associated with BOP exposure, including hypertension, ^11 , 22^ insomnia, ^23 , 24^ noise-induced hearing injury (NIHI), ^25^ depressive symptoms, ^26^ and migraine headache. ^27^

Due to logistical, safety, and ethical constraints in studying blast-related injuries, the evidence base regarding long-term health effects of BOP exposure remains limited. Clarifying these associations is important for active duty military personnel, given the implications for readiness, return-to-duty decisions, career longevity, and long-term health care burden within the Military Health System. Enhanced surveillance and early intervention of at-risk individuals may support force health protection efforts.

The aims of this analysis were to compare ACSMs who served in occupations at high risk for BOP exposure with those who never served in such occupations according to: 1) demographic and military service characteristics, 2) 2024 health care provision distribution by disease burden, 3) incidence of TBI, insomnia, migraine headache, depressive or anxiety disorders, NIHI, and essential hypertension, and 4) incidence of these conditions by cumulative time since entering the cohort or occupation.

## Methods

### Data source

This retrospective cohort study received a non-human subject research determination by the Defense Health Agency Component Office of Human Research Protections (protocol 25-22055). Medical encounter and demographic data were drawn from the Defense Medical Surveillance System (DMSS), the central repository of longitudinal medical surveillance data on U.S. military members. Medical record diagnoses of deployed service members were obtained from the Theater Medical Data Store (TMDS), which is integrated within DMSS. Complete inpatient encounter data were available from 1990 through 2024; outpatient encounter data were available from 1997 through 2024; TMDS data were available from 2008 through 2024; Army personnel data were available from 1985 through 2024; Air Force and Marine Corps personnel data were available from 1990 through 2024; and deployment data were available from 1990 through 2022.

### Study population

The study population included ACSMs in the U.S. Army, Air Force, and Marine Corps who entered initial active component service from 2001 through 2024. Occupations at high risk for BOP exposure were identified using service-specific PMOS codes documented in the August 8, 2024 Deputy Secretary of Defense Memorandum. ^28^ ACSMs were assigned to the high-risk BOP cohort at the time they first entered 1 of these occupations on or before December 31, 2024. ACSMs assigned to all remaining occupational specialties defined the cohort not considered to be at high risk for occupational BOP exposure (referred to as “low-risk” occupational cohort). ACSMs in the Navy were excluded entirely due to incomplete occupational data in DMSS.

For the aim 2 burden analysis, medical encounters occurring in calendar year (CY) 2024 among individuals in the study population were summarized according to the primary (first-listed) diagnosis using International Classification of Diseases, 10th Revision (ICD-10) codes (A00–T88, Z37, U07.1, U09) and DOW unique personal history codes (DOD0101–DOD0105). This limited, cross-sectional sampling period was specifically chosen to appraise current force readiness and the contemporary health care burden on the Military Health System, rather than to provide a cumulative representation of the historical cohort’s longitudinal morbidity. Diagnoses were classified into 25 major disease categories and 157 subcategories using the *MSMR* burden analysis methodology, a modified version of the World Health Organization Global Burden of Disease framework. ^29 , 30^ Burden estimates included the number of encounters (limited to 1 encounter per person per category per day, with inpatient encounters prioritized over outpatient encounters) and hospital bed days derived from inpatient encounters.

For aims 3 and 4, the incidence of TBI, insomnia, migraine headache, depressive or anxiety disorders, and NIHI were assessed using all available records and ICD-9 and ICD-10 diagnosis codes defined by corresponding Armed Forces Health Surveillance Division case definitions. ^31 - 36^ For migraine headache, menstrual migraine diagnosis codes were excluded. Incident cases of essential hypertension were identified using ICD-9 (401*) and ICD-10 (I10.0, I16.0*) diagnosis codes (asterisks denote inclusion of all subsequent digits or characters within the specified code). For insomnia and depressive or anxiety disorders, a qualifying case required 2 or more inpatient, outpatient, or TMDS encounters containing a case-defining diagnosis code in any diagnostic position within a 90-day period, excluding encounters occurring on the same day. For the remaining conditions, a single inpatient, outpatient, or TMDS encounter with a case-defining diagnosis code was sufficient to qualify as an incident case.

Person-years (p-yrs) of follow-up accrued from the date an ACSM entered a high-risk BOP occupation (high-risk cohort) or entered active component service (low-risk cohort) and continued until the first incident diagnosis of the outcome of interest or the end of the surveillance period (December 31, 2024), whichever occurred first. For ACSMs in the high-risk BOP cohort, person-time accrued prior to entry into a high-risk occupation was excluded. Once an ACSM entered a high-risk occupation, all subsequent person-time was attributed to the high-risk cohort regardless of subsequent occupational changes. Time since entering the cohort was calculated as the number of years in military service following entrance into the high-risk occupational cohort, or as the number of years in military service following entrance into military service (for the low-risk occupational cohort). ACSMs who met the case definition for a given outcome before entering a high-risk occupation were excluded from analyses of that outcome. Unadjusted incidence rates (IRs) were calculated per 10,000 p-yrs of active component service. It should be emphasized that this analysis evaluated occupational assignment as a proxy for BOP exposure and did not incorporate individual measures of blast exposure intensity, frequency, or cumulative dose.

### Statistical analysis

Statistical analysis for aims 1 and 2 included descriptive statistics. For aim 3, multivariable Poisson regression models were used to estimate adjusted incidence rate ratios (aIRRs) for each health condition among ACSMs in the high-risk BOP cohort compared with those who were never assigned to high-risk occupations. Models adjusted for sex, age, race and ethnicity, service branch, rank or grade, education, time since entering the cohort, and deployment history. For aim 4, person-time and incident case counts were summarized and aggregated by covariate strata, and multivariable Poisson regression models were applied to estimate aIRRs by time since entering the cohort, separately for the high-risk and low-risk cohorts. An offset for person-time was included to account for varying follow-up time within each stratum. Models adjusted for the same covariates listed earlier. All analyses were conducted using SAS Enterprise Guide (version 8.6).

## Results

### Aim 1: Demographic and military service characteristics of study population

The Army accounted for the largest proportion of ACSMs in both occupational cohorts, although it represented a larger share of the occupational cohort at high risk for BOP exposure than the low-risk occupational cohort (68.1% vs 43.6%) **(Table 1)** . Compared with the low-risk occupational cohort, the high-risk occupational cohort included a greater proportion of male (92.2% vs 79.9%) and non-Hispanic White (68.0% vs 58.0%) ACSMs. Additionally, fewer ACSMs in the high-risk occupational cohort had not deployed by the end of CY 2022 compared to the low-risk occupational cohort (55.4% vs 64.5%). In both cohorts, most ACSMs were enlisted (92.6% in high-risk cohort, 91.0% in low-risk cohort) and had served between 1 and less than 6 years (62.9% and 55.3%, respectively). Among ACSMs in high-risk occupations, 59.8% were in combat-related roles, most commonly infantry-man (Army), rifleman (Marine Corps), and security forces journeyman (Air Force). In contrast, only 5.8% of ACSMs in the low-risk occupational cohort were in combat related occupations. The most common occupational categories were repair and engineering (28.9%) and communications and intelligence (26.0%).

**TABLE 1.** Demographic and Military Service Characteristics of Occupations with High and Low Risks for Blast Overpressure Exposure, Active Component, U.S. Armed Forces Who Joined Service 2001–2024

Characteristic	High-Risk Occupation		Low-Risk Occupation	
	No.	%	No.	%
Total	831,050	27.2	2,229,594	72.8
Sex				
Male	765,876	92.2	1,781,893	79.9
Female	65,174	7.8	447,701	20.1
Age group at end of follow-up, *y*				
<20	56,202	6.8	217,992	9.8
20–24	399,202	48.0	945,956	42.4
25–29	223,878	26.9	583,130	26.2
30–34	86,430	10.4	260,477	11.7
35–39	41,523	5.0	139,825	6.3
>=40	23,815	2.9	82,214	3.7
Race and ethnicity				
White, non-Hispanic	564,877	68.0	1,293,590	58.0
Black, non-Hispanic	92,302	11.1	370,235	16.6
Hispanic	117,931	14.2	357,666	16.0
Other or unknown	55,940	6.7	208,103	9.3
Branch of service				
Army	565,847	68.1	971,648	43.6
Air Force	105,383	12.7	670,896	30.1
Marine Corps	159,820	19.2	587,050	26.3
Rank (grade) at end of follow-up				
Enlisted (E1-E9)	769,511	92.6	2,029,356	91.0
Commissioned officer (O1-O10)	57,059	6.9	184,852	8.3
Warrant officer (W)	4,480	0.5	15,386	0.7
Occupation				
Combat-related	497,109	59.8	128,483	5.8
Motor transport	1,650	0.2	95,502	4.3
Pilot, air crew	3,262	0.4	50,001	2.2
Repair, engineering	64,715	7.8	644,804	28.9
Communications, intelligence	81,365	9.8	578,907	26.0
Health care	4,386	0.5	190,613	8.6
Other	178,563	21.5	541,284	24.3
Cumulative time since entering the cohort, at end of follow-up, *y*				
<1	112,115	13.5	376,406	16.9
1 to <6	522,329	62.9	1,232,985	55.3
6 to <10	108,251	13.0	328,549	14.7
>=10	88,355	10.6	291,654	13.1
Deployment history at end of follow-up (through December 2022)				
Never deployed	460,181	55.4	1,437,738	64.5
1 prior deployment	209,142	25.2	433,661	19.5
>=2 prior deployments	161,727	19.5	358,195	16.1

Abbreviation: No., number; BOP, blast overpressure.

### Aim 2: Comparison of health care provision distribution by cohort

During CY 2024, ACSMs in occupations at high risk for BOP exposure accounted for 22.2% of the 9,768,558 inpatient and outpatient medical encounters documented among the study population. The distribution of morbidity burden categories was generally similar between occupational cohorts. For both cohorts, the 5 most common major burden categories included injury and poisoning, musculoskeletal diseases, mental disorders, ill-defined signs and symptoms, and neurological conditions. Likewise, the most frequently represented subcategories were other back problems, knee injuries, arm and shoulder injuries, all other signs and symptoms, and organic sleep disorders **(Figure 1)** . A total of 270,135 hospital bed days were recorded for the study population during CY 2024, of which 24.4% occurred among ACSMs in high-risk occupations. Mental health conditions accounted for approximately 53% of hospital bed days in both cohorts **(Figure 2)** . Differences were noted in the secondary contributors to hospitalization burden. Among ACSMs in high-risk occupations, injury and poisoning (14.7%) accounted for a greater proportion of hospital bed days than maternal conditions (7.4%). In contrast, among ACSMs in low-risk occupations for BOP exposure, maternal conditions (15.4%) accounted for a larger proportion of hospital bed days than injury and poisoning (10.1%). Over-all, health care provision patterns were broadly similar between cohorts, although injury-related conditions contributed a larger share of hospitalization burden among ACSMs in high-risk occupations.

**FIGURE 1.**
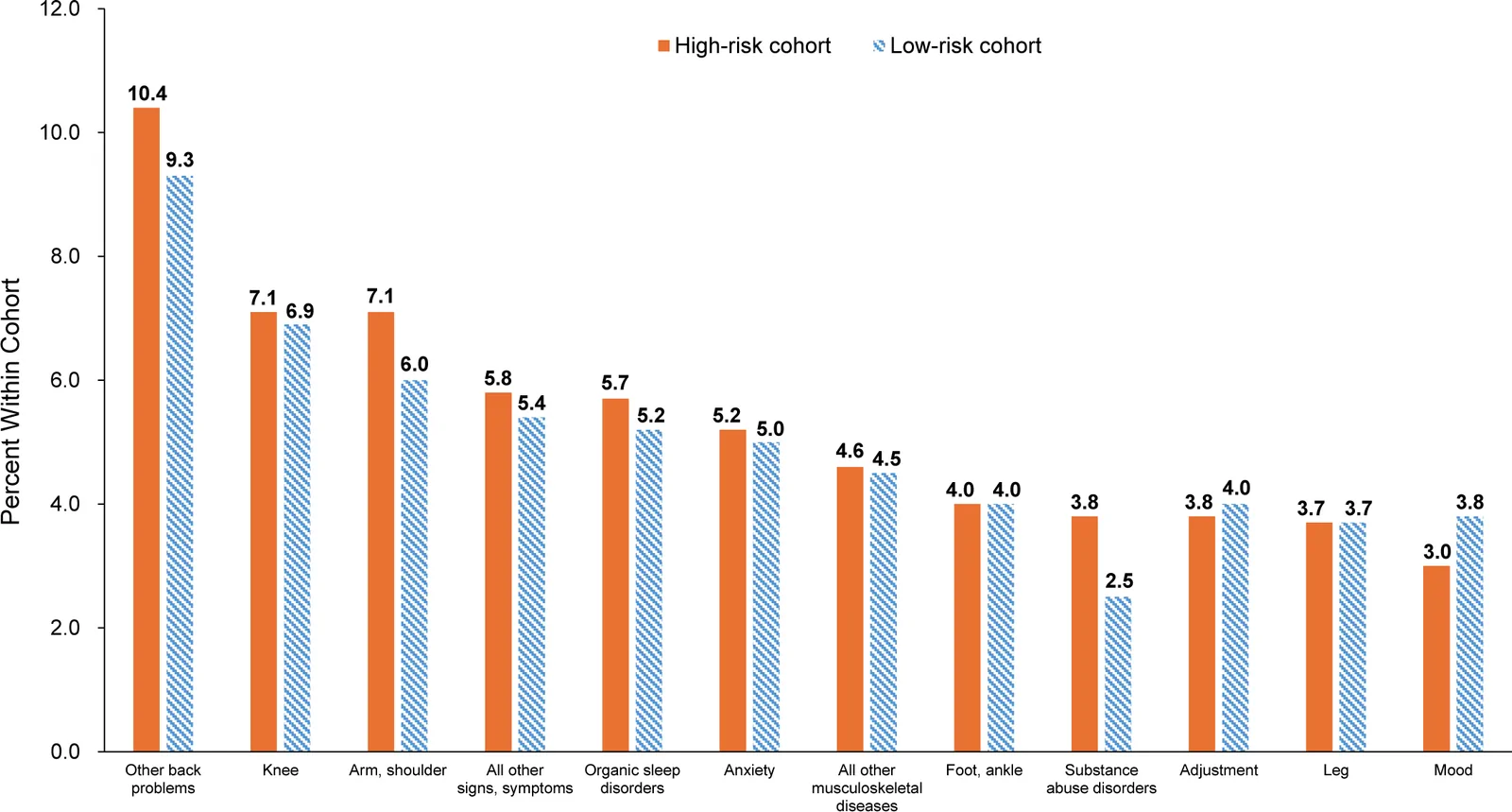
Percent Distribution of Medical Encounters by Disease Burden Subcategory in High-Risk Versus Low-Risk Blast Overpressure Occupational Cohorts, Active Component, U.S. Armed Forces, Calendar Year 2024

**FIGURE 2.**
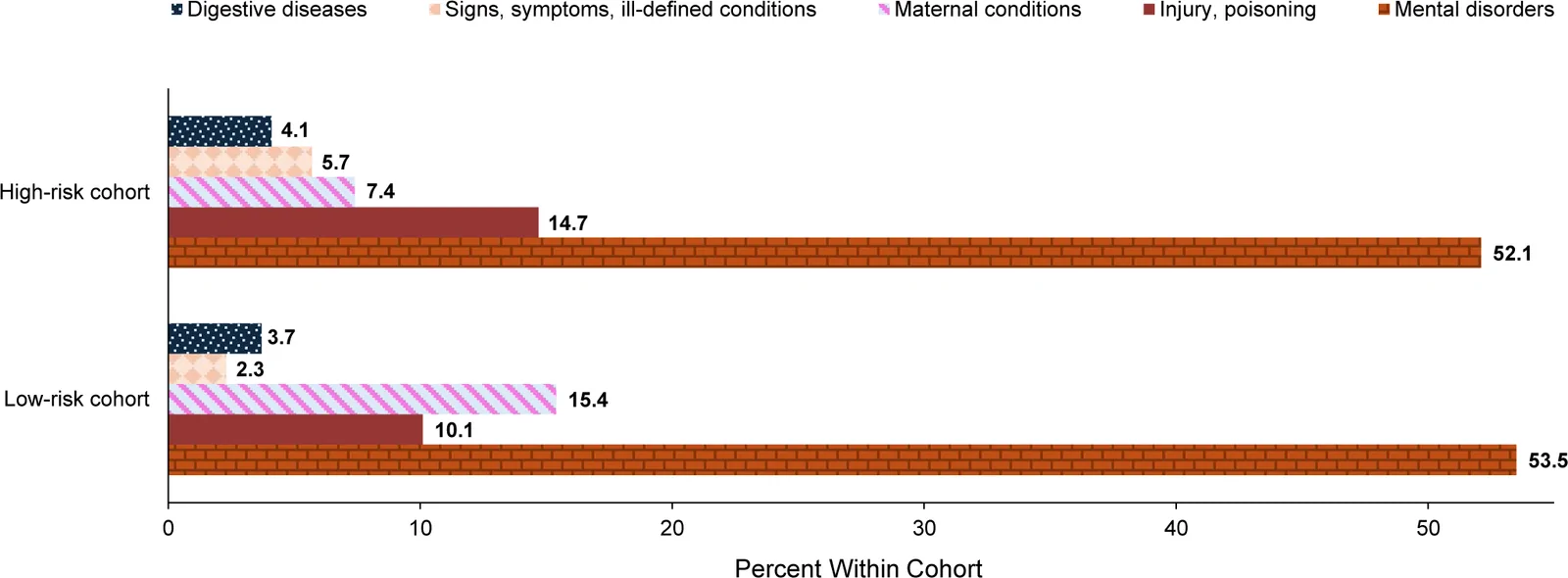
Percent Distribution of Hospital Bed Days by Major Disease Category in High-Risk Versus Low-Risk Blast Overpressure Occupational Cohorts, Active Component, U.S. Armed Forces, Calendar Year 2024

### Aim 3: Incidence of medical conditions by cohort

The adjusted incidence of essential hypertension (aIRR 0.93; 95% CI 0.92, 0.94), depressive or anxiety disorders (aIRR 0.94; 95% CI 0.94, 0.95), and migraine headache (aIRR 0.94; 95% CI 0.93, 0.95) were slightly lower in the high-risk occupational cohort compared with the low-risk cohort **(Table 2)** . Incidence of insomnia was comparable between groups (aIRR 1.01; 95% CI 1.00, 1.02). In contrast, the high-risk BOP cohort demonstrated a 39% higher adjusted incidence of NIHI (aIRR 1.39; 95% CI 1.38, 1.40) and 43% higher adjusted incidence of TBI (aIRR 1.43; 95% CI 1.42, 1.44) compared with the low-risk cohort.

**TABLE 2.** Unadjusted and Adjusted Incidence Rate Ratios for Six Medical Conditions, High-Risk Versus Low-Risk Blast Overpressure Occupations, Active Component, U.S. Armed Forces, January 1990–December 2024

	High-Risk Occupation			Low-Risk Occupation			Adjusted IRR	Adjusted 95% CI
Medical Condition	Incident Case Count	Person-Years	Incidence Rate	Incident Case Count	Person-Years	Incidence Rate		
Traumatic brain injury	88,973	3,649,972.0	243.8	154,924	10,992,928.8	140.9	1.43	1.42–1.44
Insomnia	66,680	3,801,029.2	175.4	170,280	11,104,851.6	153.3	1.01	1.00–1.02
Migraine headache	50,342	3,816,278.4	131.9	182,547	10,967,160.2	166.5	0.94	0.93–0.95
Depressive or anxiety disorders	126,790	3,684,427.4	344.1	385,147	10,631,003.6	362.3	0.94	0.94–0.95
Noise-induced hearing injury	121,666	3,549,979.9	342.7	235,986	10,820,896.4	218.1	1.39	1.38–1.40
Essential hypertension	43,983	3,821,276.1	115.1	131,766	11,093,650.0	118.8	0.93	0.92–0.94

Abbreviations: IRR, incidence rate ratio; CI, confidence interval. Incidence rate per 10,000 person-years.

### Aim 4: Incidence of medical conditions stratified by time since entering the cohort

Using less than 1 year time-in-service as the reference category for descriptive comparison of aIRRs within each occupational cohort, increased time-in-service was generally associated with increased incidence of all 6 health conditions **(Table 3)** . Within the 6-10 years and 10 years or greater time-in-service categories, aIRRs for migraine headache were higher in the high-risk occupational cohort than in the low-risk occupational cohort. Among individuals in the high-risk occupational cohort, those with 10 or more years of service had an 83% higher incidence of migraine headache compared to those with less than 1 year of service (aIRR 1.83; 95% CI 1.74, 1.93). In the low-risk cohort, the corresponding increase was 61% (aIRR 1.61; 95% CI 1.56, 1.65). In contrast, when compared descriptively, aIRRs for essential hypertension, insomnia, NIHI, TBI, and depressive or anxiety disorders were similar or lower in the high-risk occupational cohort compared with the low-risk cohort within the same time-in-service categories. Because these comparisons are descriptive, no inference should be made regarding the differential effects of cumulative BOP exposure between cohorts.

**TABLE 3.** Unadjusted and Adjusted Incidence Rate Ratios for Six Conditions by Time in Service, High-Risk Versus Low-Risk Blast Overpressure Occupations, Active Component, U.S. Armed Forces, January 1990–December 2024

		High-Risk Occupations		Low-Risk Occupations	
Medical Condition	Cumulative Time in Service	Adjusted IRR	Adjusted 95% CI	Adjusted IRR	Adjusted 95% CI
Traumatic brain injury	<1 year	Ref	—	Ref	—
	1 to <6 years	1.19	1.16–1.21	1.37	1.35–1.40
	6 to <10 years	1.20	1.16–1.24	1.42	1.38–1.46
	>=10 years	1.30	1.25–1.36	1.42	1.37–1.47
Insomnia	<1 year	Reference	—	Reference	—
	1 to <6 years	2.28	2.20–2.35	2.42	2.37–2.47
	6 to <10 years	2.70	2.59–2.82	2.89	2.81–2.96
	>=10 years	2.59	2.47–2.72	2.90	2.82–2.99
Migraine headache	<1 year	Reference	—	Reference	—
	1 to <6 years	1.46	1.41–1.51	1.48	1.45–1.50
	6 to <10 years	1.72	1.65–1.80	1.62	1.58–1.65
	>=10 years	1.83	1.74–1.93	1.61	1.56–1.65
Depressive or anxiety disorders	<1 year	Reference	—	Reference	—
	1 to <6 years	1.25	1.23–1.27	1.43	1.41–1.44
	6 to <10 years	1.51	1.46–1.55	1.70	1.67–1.73
	>=10 years	1.45	1.40–1.50	1.75	1.72–1.79
Noise-induced hearing injury	<1 year	Reference	—	Reference	—
	1 to <6 years	1.74	1.70–1.78	1.79	1.77–1.82
	6 to <10 years	1.78	1.73–1.83	1.88	1.83–1.92
	>=10 years	1.80	1.74–1.87	1.99	1.94–2.04
Essential hypertension	<1 year	Reference	—	Reference	—
	1 to <6 years	1.53	1.48 - 1.59	1.49	1.45 - 1.52
	6 to <10 years	1.61	1.54 - 1.69	1.50	1.46 - 1.54
	>=10 years	1.55	1.46 - 1.63	1.45	1.41 - 1.50

Abbreviations: IRR, incidence rate ratio; CI, confidence interval.

## Discussion

This large retrospective cohort study evaluated health outcomes among ACSMs in a high-risk occupational cohort for BOP exposure versus a low-risk cohort and identified several notable differences. Most prominently, ACSMs assigned to the high-risk cohort experienced significantly higher incidence of TBI and NIHI. These findings are consistent with the growing body of literature on occupational blast exposure risk to the central nervous and auditory systems, ^37 , 38^ including a 10-year service-wide cohort study of occupational low-level blast exposure risk. ^39^

Although individual BOP exposure was not directly measured in this study, the observed associations between incidence of TBI and NIHI and assignment to occupations at high risk for BOP exposure support continued evaluation of occupational hazard mitigation strategies, including blast-attenuating helmets and dual-layer hearing protection. Together with prior evidence supporting the biological plausibility of blast-related neurological and auditory injury, these results reinforce the potential value of periodic neurocognitive and audiologic screening among personnel in high-risk occupational specialties, including infantry, artillery, and combat operations. Finally, the higher incidence of TBI and NIHI in the high-risk occupational cohort may be multifactorial and reflect additional characteristics of these occupations, including differences in physical demand, environmental exposures, training activities, and medical surveillance. Future studies incorporating more detailed occupational and individual exposure data are needed to better clarify the contributions of these factors.

Conversely, adjusted analyses demonstrated similar or lower incidence of essential hypertension, migraine headache, depressive or anxiety disorders, and insomnia in the high-risk cohort compared with the low-risk cohort. This seemingly protective pattern may reflect the ‘healthy warrior effect,’ whereby individuals in combat-related occupations are generally healthier and more physically fit. These occupations also impose strict accession and retention standards, which may result in selection of healthier individuals for entry and medical attrition or reclassification of those who develop health conditions. Lower observed incidence of mental health disorders may also be influenced by under-diagnosis or under-reporting due to stigma and barriers to seeking care. Additionally, individuals with genetic predisposition for mental health and neurological conditions may exclude or remove themselves from physically demanding occupations that could accelerate or exacerbate symptoms. Residual confounding differences in health care provision between occupational cohorts may also have contributed to these findings. Given that several estimates were close to the null, the clinical significance of these differences should be interpreted cautiously.

The time-in-service analysis identified differences in aIRRs among cumulative time-in-service categories within each occupational cohort. For most conditions, aIRRs were stable or increased with progressive time since entering the study for both cohorts, with the highest estimates observed among individuals with 10 or more years of service. For migraine headache, however, aIRRs were consistently higher among cumulative time-in-service categories in the high-risk occupational cohort compared to the low-risk occupational cohort. These findings are compatible with a cumulative exposure hypothesis but should be interpreted cautiously, as cumulative time-in-service is not a direct measure of cumulative BOP exposure. In contrast, aIRRs for TBI were consistently lower for the same time-in-service category in the high-risk occupational cohort compared to the low-risk occupational cohort. This finding was unexpected and warrants further investigation. Since this analysis focused on first-ever TBI diagnoses, results may differ if recurrent TBI or subclinical injuries were included. Future studies incorporating individual-level blast exposure metrics, including blast frequency, estimated overpressure, average and cumulative psi from dosimetry readings, and use of protective equipment, are needed to directly evaluate cumulative BOP exposure and better characterize exposure and outcome relationships.

The morbidity burden analysis demonstrated broadly similar patterns of health care provision among cohorts, despite differences in sex, race and ethnicity, service branch, occupational code, and deployment history. In both groups, health care provision was primarily driven by injuries, musculoskeletal conditions, mental health disorders, and neurological conditions. These patterns suggest that occupational BOP exposure may not substantially alter overall patterns of health care, although subtle differences in specific diagnostic categories were observed. Maternal conditions accounted for a higher proportion of hospital bed days in the low-risk occupational cohort compared to the high-risk cohort (15.4% vs. 7.4%, respectively), likely secondary to the higher proportion of women in this cohort. Interestingly, substance use disorders accounted for a greater proportion of outpatient encounters (3.8% vs. 2.5%) and hospital bed days (17% vs. 13.9%) in the high-risk cohort compared to the low-risk cohort. These findings emphasize the importance of continued evaluation of behavioral health outcomes in populations with potential occupational blast exposure.

There are several limitations to this study. First, ACSMs in the Navy, the second largest branch of the U.S. Armed Forces (n=337,800), ^40^ were excluded due to incomplete occupational data in DMSS, limiting generalizability for the total force.

Second, the absence of individual data on BOP exposure characteristics (e.g., frequency, intensity, protective equipment use) likely resulted in exposure misclassification. Our use of a static exposure classification—whereby individuals were permanently assigned to the high-risk cohort upon their first entry into a high-risk occupation—did not account for subsequent changes in occupational risk. For instance, a service member who spent only a few years in a high-risk occupation before transferring to a low-risk role was still attributed entirely to the high-risk cohort. This exposure misclassification dilutes the true effect of continuous exposure, biasing the incidence rate ratios toward the null. Consequently, the estimates reported in this study likely underestimate the true magnitude of differences in disease incidence between the high-risk and low-risk occupational groups.

Third, outcome detection bias may have occurred since ACSMs in high-risk occupations may undergo more frequent occupational health evaluations. Additionally, follow-up for the high-risk cohort began at the time of occupational entry rather than entry to service, resulting in a short period of military service prior to entering the cohort (median 30 days, mean 144 days). While this slight misalignment in follow-up start times could introduce a survivor effect, sensitivity testing adjusting for time since military entry demonstrated minimal impact on the aIRRs, indicating that the overall findings remain robust.

Fourth, this analysis did not perform exposure lag modeling to account for latency periods in conditions with delayed onset, such as essential hypertension and NIHI. Additionally, study outcomes were identified using medical encounter diagnosis codes and may not capture all underlying symptoms or diseases. Finally, behavioral and lifestyle factors (e.g., tobacco use, alcohol consumption, sleep, physical fitness) were not available and may confound or modify exposure and outcome relationships.

This analysis contributes to the growing evidence that links service in occupations at high risk for BOP with adverse health outcomes. Importantly, it complements, rather than replaces, direct individual exposure monitoring, which remains essential for accurately characterizing cumulative blast exposure. These findings support continued surveillance, targeted prevention efforts, and enhanced exposure monitoring for personnel in high-risk occupations to better define the long-term health effects o f b last e xposure, i nform force health protection strategies, and preserve military readiness.
